# Inhaled Corticosteroids Use Is Not Associated With an Increased Risk of Pregnancy-Induced Hypertension and Gestational Diabetes Mellitus: Erratum

**DOI:** 10.1097/MD.0000000000007016

**Published:** 2017-05-19

In the article, “Inhaled Corticosteroids Use Is Not Associated With an Increased Risk of Pregnancy-Induced Hypertension and Gestational Diabetes Mellitus”,^[1]^ which appeared in Volume 95, Issue 22 of *Medicine*, two authors’ affiliations appeared incorrectly. Eun Jin Janh's affiliation also includes “Department of Information Statistics, Colloge of Natural Science, Andong National University, Andong, Republic of Korea.” Jimin Kim's affiliation should have included “’Department of Health Policy and Hospital Management, Graduate School of Public Health, Korea University, Seoul, Republic of Korea.”
